# The koala (*Phascolarctos cinereus*) prostate: a comprehensive histological and immunohistochemical investigation

**DOI:** 10.1093/biolre/ioad098

**Published:** 2023-08-18

**Authors:** Yolande Campbell, Chiara Palmieri, Sara Pagliarani, Jo Gordon, Stephen Johnston

**Affiliations:** School of Environment, The University of Queensland, Gatton, 4343, Australia; School of Veterinary Science, The University of Queensland, Gatton, 4343, Australia; School of Veterinary Science, The University of Queensland, Gatton, 4343, Australia; School of Veterinary Science, The University of Queensland, Gatton, 4343, Australia; School of Veterinary Science, The University of Queensland, Gatton, 4343, Australia; School of Environment, The University of Queensland, Gatton, 4343, Australia; School of Veterinary Science, The University of Queensland, Gatton, 4343, Australia

**Keywords:** prostate, koala, marsupial, male reproductive, prostasomes, koala reproduction phascolarctos cinereus, immunohistochemistry, comparative anatomy

## Abstract

The prostate of the koala (Phascolarctos cinereus), and of marsupials more generally, is the primary contributor of seminal fluid, yet comparatively little is known about its microanatomy or biochemistry. This study explored evidence of parenchymal segmentation of the koala prostate. The prostate of three sexually mature koalas were processed for histopathology, histochemistry (Masson’s trichrome, Alcian Blue, periodic acid Schiff staining), and immunohistochemistry using basal (tumor protein 63, cytokeratin 14) and luminal (cytokeratin 8/18, prostate specific antigen, androgen receptor) markers. Results confirmed clear segmentation of the koala prostate into three zones, anterior, central, and posterior, characterized by differences in the proportion of glandular tissue, as well as the thickness of collagen fibers; there were also distinct differences in the secretions produced in each zone. Based on immunohistochemistry, the koala prostate showed evidence of both basal proliferative and luminal secretory cells. The ratio of cell types varied across the three segments, with the central segment housing the highest density of basal cells. Globular bodies produced in the anterior zone were shown to possess the same markers as those described for human prostasomes. This study is the first to comprehensively document the marsupial prostate in terms of microanatomy and corresponding immunohistochemistry. While further biochemical analysis, such as proteomics of each segment will better define the relative functions of each tissue, the data presented here are consistent with the hypothesis that the koala prostate potentially represents an example of an ontological stage in the evolutionary differentiation of male eutherian accessory glands.

## Teaser

This is the first study to comprehensively document a marsupial prostate in terms of microanatomy and corresponding immunohistochemistry.

## Graphical Abstract

**Graphical Abstract:**
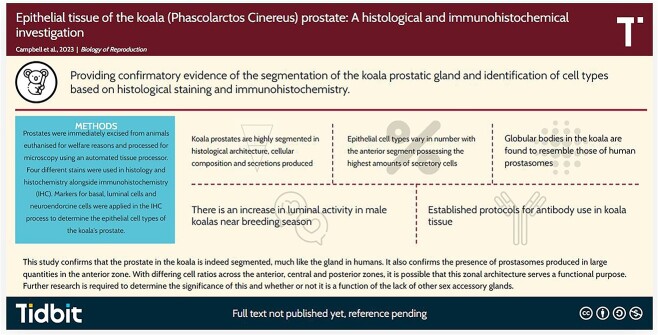

## Introduction

The prostate is an accessory sex gland of male mammals with a pivotal role in the production of seminal fluids required for successful reproduction. In male marsupials, the prostate is a prominent feature and in some species can be the largest glandular structure during times of peak activity [1, 2]. Despite this, the structure and function of the prostate in this taxon remains poorly understood; most of the literature is outdated or lacks sufficient morphological detail (see Table 1). As the prostate is hypothesized to be the primary source of seminal fluid in the koala (*Phascolarctos cinereus*) [3], its importance in sperm physiology and transport requires further study, as does its potential role in inducing ovulation [4]. Given the endangered conservation status of this species [5], assisted breeding technology [6] may be required in the future, and as such, a greater understanding of their reproductive organs is imperative. Furthermore, a more thorough description and understanding of the koala prostatic tissue should also provide a baseline for assessing the adverse effect of *Chlamydia*-induced pathology on the prostate [7, 8].

**Table 1:** Historical publications of normal prostatic glands in koalas to date.

Author and year of publication	Methodology	Animals studied
Young (1879)[44]	Gross anatomy	1
Temple-smith and Taggart (1990)	Histology, TEM	3
Larkin et al. (2017)[45]	Ultrasound	62
Skerrett-Byrne et al. (2021)	Proteomics	4
Allen et al. (2010)	Gross anatomy	95
Pagliarani (2021)	Histology, histochemistry, IHC	12
Palmieri et al. (2019)	Histology, histochemistry, IHC	52

TEM, transmission electron microscopy; IHC, immunohistochemistry.

The koala prostate, and that of humans, appear to share similarities, as evidenced in the first proteomic study of the composition of koala semen by Skerrett-Byrne et al. [3]. Koala prostatic bodies were shown to share 82.5% of the same proteins as human prostasomes, with the majority of proteins in both species being enzymatic, with an overrepresentation of molecular chaperone and stress proteins [3].

Previous histological descriptions of the koala prostate [2] and those of the human prostate [9] reveal that both species present with a well-defined zonal architecture, the significance of which is not yet known. In the koala, and other marsupials, Rodger and Hughes [10] have suggested that this segmentation likely arises as a result of the specialization of the prostate to produce different constituents of seminal plasma. Furthermore, apocrine secretory, globular bodies present in the koala prostatic and seminal fluid potentially have a similar appearance to that of human prostasomes (extracellular vesicles released by the prostate epithelium) in terms of their morphology, potential function, and protein composition [3]. The topic of prostasomes and their role in fertility is a focus of much scientific interest in the field of human reproductive medicine [11] and the koala prostate could play a role in this context. Hence, an improved characterization of koala prostatic structure and function would not only lead to a more comprehensive understanding of seminal physiology and prostatic disease but potentially also serve as an animal model for studies of human prostatic biology.

The underlying hypothesis of this study was that the zonal anatomy of the adult koala prostate gland can be clearly delineated on the basis of microanatomy and immunohistochemistry.

## Materials and methods

### Animals and tissue collection

Prostatic tissues used in this study were obtained from sexually mature male koalas presented to wildlife hospitals in Southeast Queensland. All animals were euthanized for welfare reasons and the glands were excised within the hour. Koala #1 had deteriorated because of osteosarcoma and presented with low body condition and, as such, was euthanized 15 September 2020; koala #2 had moderate body condition and was euthanized 12 May 2022 because of vehicular trauma; whereas koala #3, sourced on 26 July 2022, was adult with moderate body condition that was euthanized following a dog attack. All koalas used in this study presented with no clinical signs of active reproductive tract chlamydiosis and were tested for urogenital chlamydiosis by means of PCR [12] or loop-mediated isothermal amplification without nucleic acid purification [13] and displayed negative results. This study was approved by the University of Queensland Animal Ethics Committee (Permits 2021/AE00108 and ANRFA/399/19). The entire prostate gland in each koala was excised cranially at the base of the bladder, with the caudal margin defined as the distal boundary of the membranous urethra. Tissues were fixed in 10% neutral buffered formalin for a minimum of 48 h, washed in phosphate buffered saline (pH 7.2), and stored in 70% ethanol prior to histological processing.

### Tissue processing, histology, and immunohistochemistry

Each prostate sample was trimmed, placed in histology tissue-cassettes, and set for standard overnight processing using an automated tissue processor (Tissue Tek VIP 6 AI-E2; Sakura, Netherlands). This included dehydration of tissue in a series of alcohol stations and a final exposure to a series of xylene stations. The tissues were embedded in paraffin, blocks sectioned at 4 μm, and mounted onto slides for hematoxylin and eosin (H&E) staining, other histochemical staining, or immunohistochemical processing. One longitudinal section per animal was prepared for analysis. The histochemical stains included (1) Masson’s trichrome to delineate connective tissue/collagen [14], (2) periodic acid Schiff (PAS) to detect polysaccharides (glycoproteins, glycogen, glycolipids), and (3) Alcian blue to identify acidic epithelial and connective tissue mucins [15]. Staining was conducted using a combination of an autostainer (ST5020; Leica Australia), but with the addition of manual processing for Masson’s trichrome, PAS, and Alcian blue. Following staining, a water-free, polymer mounting media (Entellan New; Merck, Germany) was used to cover the slides.

Antibodies employed in the current study for immunohistochemistry included cytokeratin 14 (CK14) and tumor protein 63 (p63) for basal cells, and cytokeratin 8/18 (CK8/18), prostate-specific antigen (PSA) and androgen receptor (AR) for luminal cells (Table 2). These antibodies have been established in our laboratory as validated markers for normal and neoplastic prostatic cells in dogs [16]. All antibodies were raised against human target proteins, and as such, an evaluation of the extent of protein homology with koala tissue was conducted using NCBI protein BLAST prior to IHC to determine if there would be sufficient cross-reactivity [17]; specific details of this analysis are available from the corresponding author. All antibodies were run in-house through an automated IHC staining process using various dilutions on control tissue to optimize the dilution ratio for each antibody. The control tissue was known to contain the relevant protein being investigated. Canine prostate was used for AR, PSA, CK8/18, and p63 and canine mammary glands were used for CK14. Negative controls (same sections without the incubation with the primary antibody) were also run alongside dilution optimizations (Table 2). Once the optimal dilutions were determined, the antibodies were processed on koala prostatic tissue alongside positive and negative controls.

**Table 2:** Antibodies, control tissues, and optimized dilution ratio.

Antibody	AR	CK8/18	CK14	p63	PSA
					
Supplier	Sigma-Aldrich	Novocastra	Thermo Scientific	Abcam	Dako
Cat. #	SAB4501575	NCL-L-5D3	MA5-11599	ab735	M0750
Target	Androgen receptor	Cytokeratin (8/18)	Cytokeratin 14 (human, mouse, rat)	p63 (human, mouse, rat)	Purified human prostate-specific antigen
Clone	SAB4501575	5D3	LL002	4A4	ER-PR8
Use in this manuscript	Detection of luminal cells	Detection of luminal cells	Detection of basal cells	Detection of basal cells	Detection of luminal cells
RRID	AB_10745900	AB_563833	AB_10982092	AB_305870	AB_2281105
Type	Rabbit polyclonal	Mouse monoclonal	Mouse monoclonal	Mouse monoclonal	Mouse polyclonal
Optimized dilution	1:150	1:200	1:800	1:50	1:2400
Control tissue	Canine prostate	Canine prostate	Canine mammary gland	Canine prostate	Canine prostate

IHC was performed using an Autostainer (LINK48; Dako, Denmark) with the PT Link Tank (PT200; Dako) for pre-treatment using high pH target retrieval. Tissues were dewaxed, rehydrated, and submerged in a pH 9 solution containing 50× concentrated Tris/EDTA (EnVision FLEX) for 20 min at 97°C. Tissues were then allowed to cool for 10 min before antibody dilutions were applied to each slide and then incubated for an hour at room temperature. Immunohistochemical staining was performed on deparaffinized sections with the horse radish peroxidase (HRP), (3,3′-diaminobenzidine) (DAB) complex procedure. Slides were incubated with peroxidase blocking reagent (a phosphate buffer containing hydrogen peroxide, 15 mmol/L NaN_3_, and detergent) for 5 min, then HRP (dextran coupled with peroxidase molecules and goat secondary antibody molecules) for 20 min, then the slides were developed with DAB + Sub-Chromo solution for 10 min. Finally, the sections were counterstained with Mayer’s hematoxylin for 5 min. Between each step, buffer solution (hydrogen peroxide and preservatives) was applied for 5 min. All IHC procedures were performed under identical conditions and all solutions used were EnVision FLEX. To compare the expression of the different markers in each of the prostatic zones, a semi-quantitative approach was used evaluating the percentage of positive cells as less than 10%, between 10 and 50%, and more than 50%. A qualitative assessment of staining intensity was also applied, expressed as mild, moderate, or strong. The analysis was performed blind by specialist veterinary pathologist CP.

## Results

### Koala prostate histology and histochemistry

**Figure 1:**
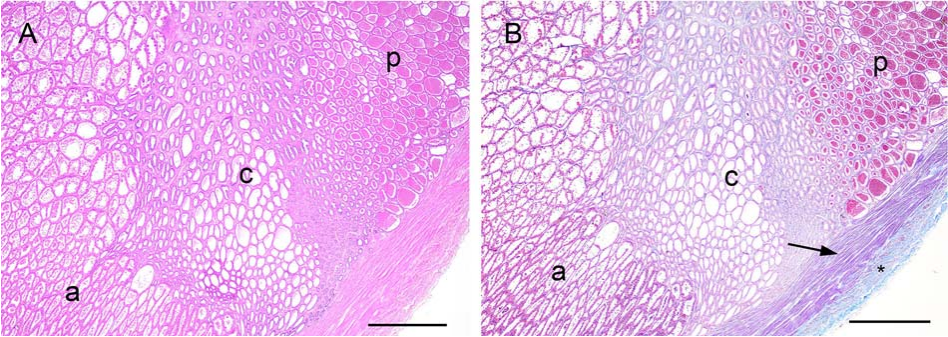
Segmentation of the koala prostate on HE-stained sections. (**A**) and Masson’s trichrome stained sections (**B**). a = anterior prostate; c = central prostate; p = posterior prostate. Note the connective tissue between the glands and in the capsule (asterisk) and the smooth muscle layer (arrow) in (B) (bar = 1000 µm).

Histologically, the carrot-shaped prostate was enclosed in a fibromuscular capsule and consisted of numerous glands radially arranged toward the central prostatic urethra. H&E and Masson’s trichrome staining clearly defined the posterior, central, and anterior zones (Figure 1A and B), with the posterior segment representing up to 50% of the total prostate tissue, the central segment 15%, and the anterior segment 45%. The cellular secretions in the acini of each segment were significantly different in composition, as described in the following sections.

#### Anterior prostate

**Figure 2:**
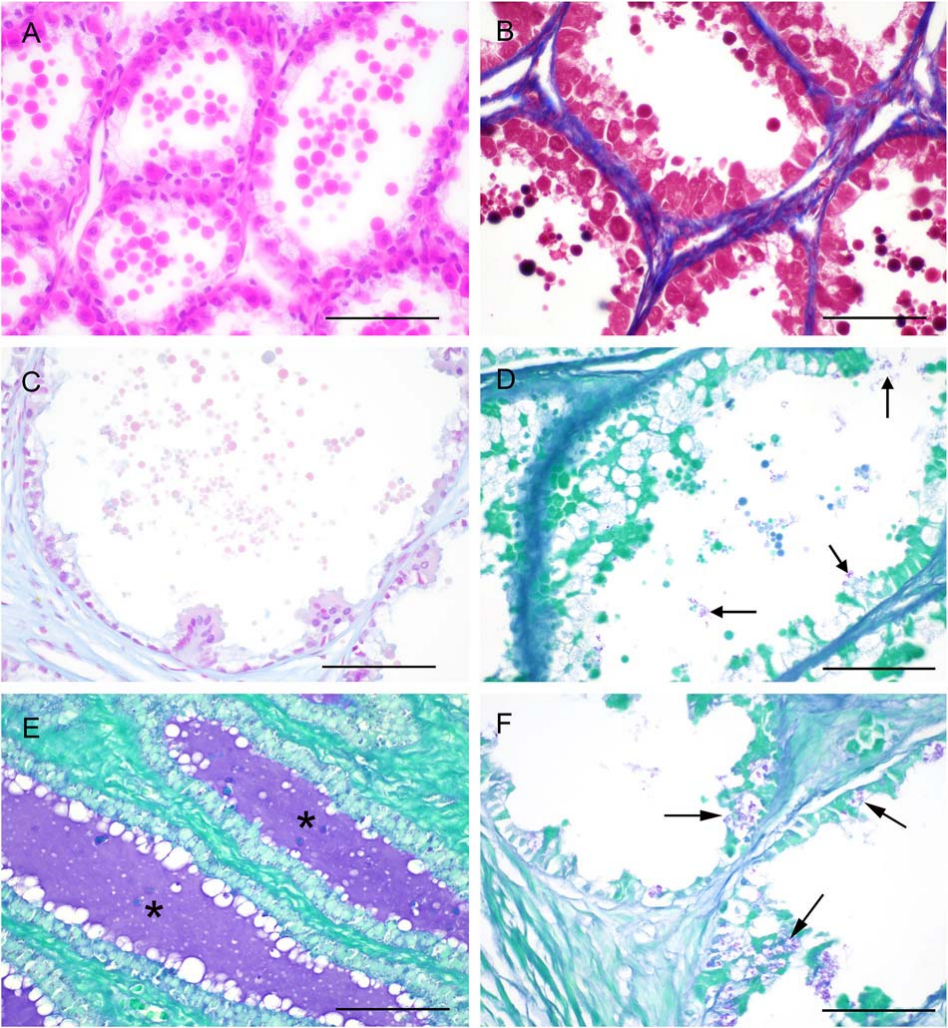
Morphological features of the anterior segment of the koala’s prostate. (**A**) Glands lined by a single layer of epithelial cells and containing hypereosinophilic secretory globules. HE (bar = 250 μm). (**B**) Scarce connective tissue (blue) within the interstitium of the anterior segment. Masson’s trichrome (bar = 250 μm). (**C**) Alcian blue-negative intraluminal secretory material (bar = 250 μm). (**D**) Occasional PAS-positive granular material within the glandular lumen (bar = 250 μm). (**E**) homogeneous PAS-positive material within the glandular lumen as indicated by the asterisk (bar = 500 μm). (**F**) PAS-positive granular material (arrows) within the cytoplasm of lining epithelial cells (bar = 250 μm).

The large tubule-alveolar glands of the anterior zone were predominately circular to oval and were surrounded by a small amount of fibromuscular stroma (Figure 2A). A distinct layer of cuboidal to columnar cells (10–15 μm in diameter) lined these glands. The epithelial lining consistent of two cell populations: (1) large, tall cuboidal to occasionally columnar eosinophilic cells with moderate amount of homogeneous hypereosinophilic cytoplasm, a central granular nucleus, and distinct cytoplasmic borders; (2) clear cuboidal cells with a basal hyperchromatic nucleus and a moderate amount of clear to vacuolated cytoplasm. The glands were filled with large volumes of hypereosinophilic globular material, appearing to be “pinching off” from the columnar cells. The inter-glandular stroma consisted of scarce Masson-positive collagen fibrils (Figure 2B). The intraluminal secretions were consistently Alcian blue negative (Figure 2C), whereas the PAS staining varied from a low number of randomly scattered globules to a diffuse staining of homogeneous secretory material. PAS-positive granules were observed in the cytoplasm of the glandular epithelial cells, although not consistently across the anterior zone (Figure 2D–F).

#### Central prostate

**Figure 3:**
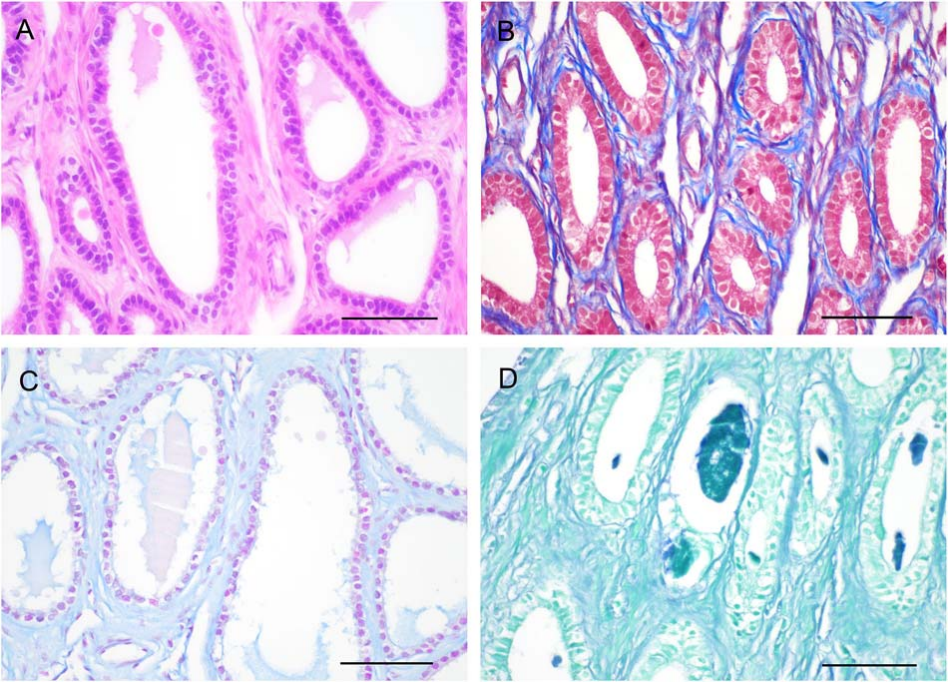
Morphological features of the central segment of the koala’s prostate. (**A**) Multiple small glands lined by a cuboidal to short columnar epithelium. HE (bar = 250 μm). (**B**) moderate amount of connective tissue (blue) interspersed between the glands. Masson’s trichrome (bar = 250 μm). (**C**) Alcian blue-negative secretory material within the lumen (bar = 250 μm). (**D**) PAS-negative secretory material within the glandular lumen (bar = 250 μm).

The glandular tissue of the central zone was composed of a combination of large, elongated tubule-alveolar glands and smaller circular glands interspersed with higher amounts of fibromuscular stroma (Figure 3A). Like the posterior segment, the glands were lined by a single, occasionally double, layer of cuboidal to short columnar cells (10–15 μm in diameter) with a low to moderate amount of eosinophilic cytoplasm. The hyperchromatic nucleus was located peripheral to basal with no notable nucleoli. Low amounts of eosinophilic homogenous material were present in approximately 50% of the acini. The thick inter-glandular stroma consisted of large bundles of Masson-positive collagen fibers (Figure 3B). The intraluminal secretions were consistently PAS and Alcian blue negative (Figure 3C and D).

#### Posterior prostate

**Figure 4:**
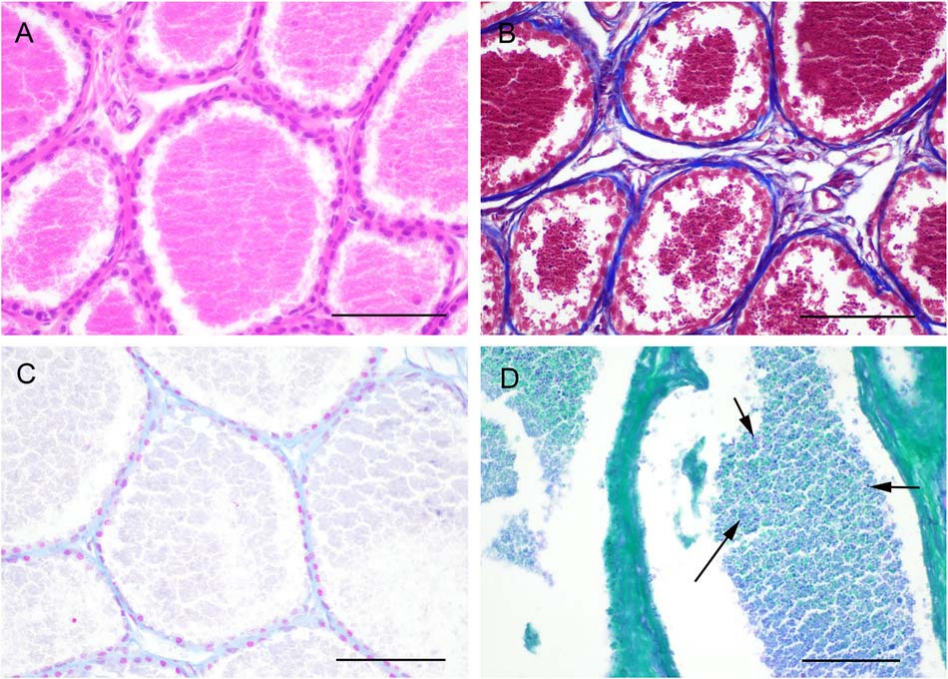
Morphological features of the posterior segment of the koala’s prostate. (**A**) Multiple glands lined by a single layer of cuboidal epithelial cells with intraluminar granular material. HE (bar = 250 μm). (**B**) Scarce amount of connective tissue (blue) separating the glands. Masson’s trichrome (bar = 250 μm). (**C**) Alcian blue-negative secretory material within the lumen (bar = 250 μm). (**D**) Randomly scattered PAS-positive granules within the lumen of the glands (arrows) (bar = 250 μm).

The posterior prostate consisted of high numbers of round to elongated tubule-alveolar glands separated by low amounts of fibrous stroma (Figure 4A). Each gland was lined by a clearly defined layer of cuboidal to short columnar cells (10–15 μm in diameter) with low to moderate amount of eosinophilic cytoplasm, a round to oval hyperchromatic peripheral to basal nucleus and absence of obvious nucleoli. The glandular lumen was filled with a moderate to high amount of eosinophilic granular material, multifocally overlying the epithelial layer. The inter-glandular stroma consisted of a Masson-positive thin layer of collagen fibers admixed with fibrocytes and small blood vessels (Figure 4B). While the intraluminal secretions were consistently Alcian blue negative (Figure 4C), there were occasional PAS-positive granules within the lumen (Figure 4D).

#### Miscellaneous histological findings

**Figure 5:**
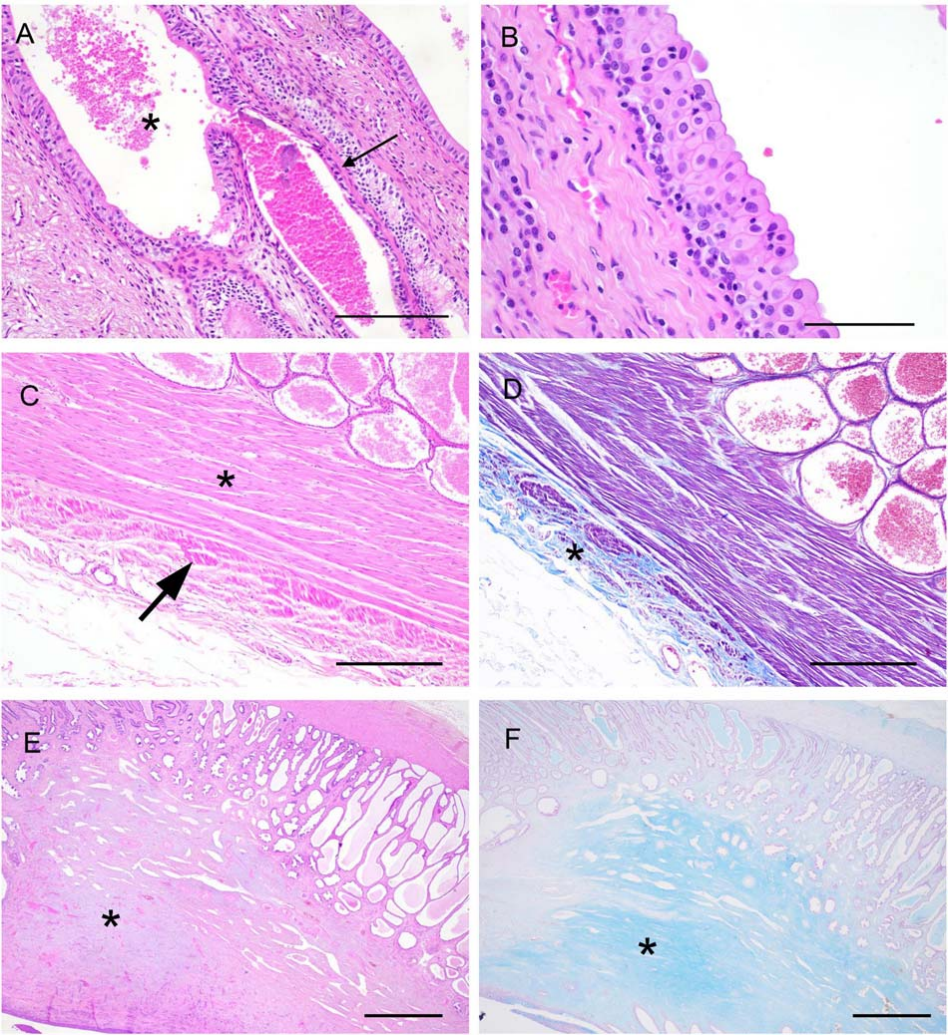
Morphological features of periurethral ducts, prostatic urethra, and stroma of the koala’s prostate. (**A**) Prostatic ducts (asterisk) lined by a pluristratified epithelium and connected to the glandular structure (arrow). HE (bar = 500 µm). (**B**) Prostatic urethra with a transitional epithelium. HE (bar = 250 µm). (**C**, **D**) Capsule of the prostate with an inner circular (asterisk) and outer longitudinal (arrow) smooth muscle layer admixed with low amount of connective tissue (asterisk in D—Masson’s trichrome). HE (bar = 1000 µm). (**E**, **F**) Myxoid stroma toward the center of the prostate (asterisk) with a strong Alcian blue-positive staining (F) HE (bar = 1000 µm).

The prostatic glands from each zone opened into periurethral ducts lined by a pluristratified columnar epithelium (Figure 5A) and connected to the central prostatic urethra which was lined by a pluristratified transitional epithelium (Figure 5B). The capsule consisted of a thick inner circular and outer longitudinal layer of smooth muscle cells with an outermost layer of connective tissue lined by mesothelial cells (Figure 5C and D). In two of the three koala prostate samples (#1 and #3), randomly scattered accumulations of pale basophilic material were interspersed among the stromal connective tissue, in particular around the prostatic urethra. The myxoid nature of the basophilic material was further confirmed by the Alcian blue staining (Figure 5E and F).

### Koala prostate immunohistochemistry

#### Anterior prostate

**Figure 6:**
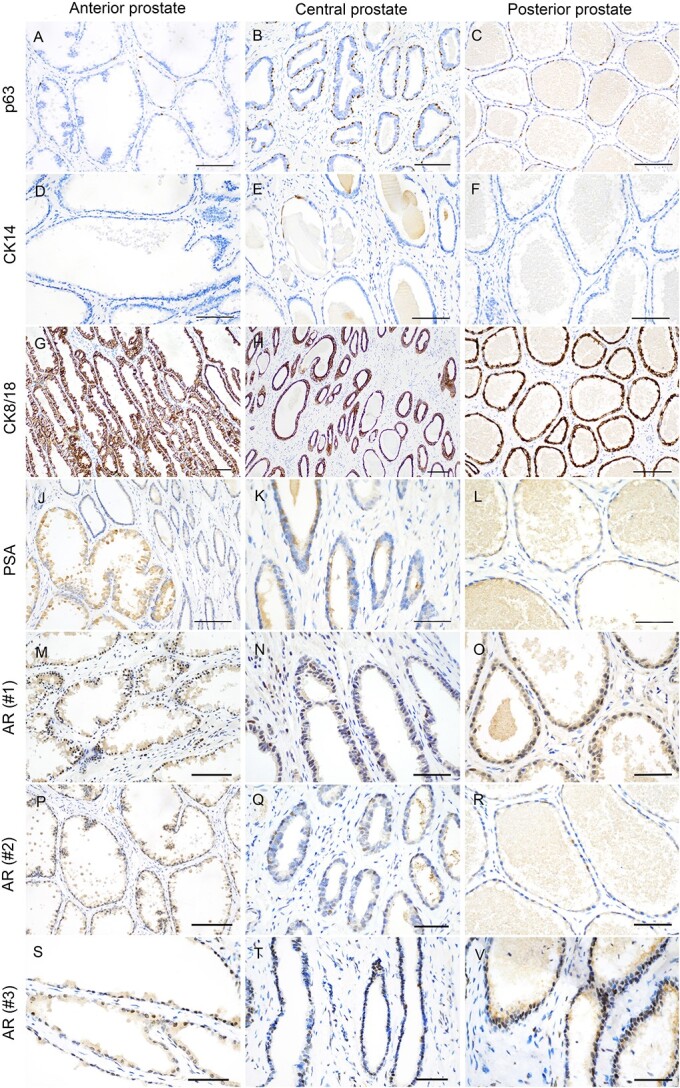
Immunohistochemical characterization of the different segments of the koala prostate. Anterior prostate: (**A**) p63 expression in only 10% of cells (bar = 100 μm). (**D**) Complete absence of CK14 expression (bar = 100 μm). (**G**) Strong cytoplasmic staining of CK8/18 in more than 80% of cells (bar = 100 μm). (**J**) PSA expression in more than 50% of cells and in the apocrine sections (bar = 100 μm). (**M**) Moderate to strong intensity of AR expression in more than 50% of cells in koala #1 (bar = 100 μm). (**P**) Moderate AR expression in more than 50% of cells in koala #2 (bar = 100 μm). (**S**) Moderate intensity of expression of AR in koala #3 (bar = 50 μm). Central prostate (**B**) P63 expression in 10–50% of cells (bar = 50 μm). (**E**) Cytoplasmic expression of CK14 in less than 10% of cells (bar = 50 μm). (**H**) Strong expression of CK8/18 in more than 50% of cells (bar = 50 μm). (**K**) Mild cytoplasmic expression of PSA in 10–20% of cells (bar = 50 μm). (**N**) More than 50% of cells expresses AR in #1 (bar = 50 μm). (**Q**) AR expression in 10% of cells in koala #2 (bar = 50 μm). (**T**) AR expression in 10–50% of cells in koala #3 (bar = 50 μm). Posterior prostate (**C**) p63 staining of basal cells (bar = 100 μm). (**F**) CK14-positive basal cells (bar = 100 μm). (**I**) CK8/18 expression in the luminal cell cytoplasm (bar = 100 μm). (**L**) Mild cytoplasmic staining of PSA in luminal cells (bar = 50 μm). (**O**) AR expression in koala #1 displaying in more than 50% of cells with strong intensity of staining (bar = 50 μm). (**R**) AR expression in 10–50% of cells in koala #2 (bar = 50 μm). (**V**) AR expression in more than 50% of cells in koala #3 (bar = 50 μm).

In the anterior zone, fewer than 10% of cells were strongly positive for p63. P63-positive cells were randomly scattered and formed a discontinuous layer close to the basement membrane. The density of p63-positive cells was lower compared with the central and the posterior zones (Figure 6A). CK14 appeared to be completely absent from this zone (Figure 6D). CK8/18 was expressed in more than 50% of luminal cells with strong cytoplasmic staining (Figure 6G). The anterior zone showed a significant amount of secretory activity in the luminal layer, with more than 50% of luminal cells expressing cytoplasmic PSA. Furthermore, the intraluminal secretory material was PSA positive with a strong intensity of expression, including the apocrine secreted globular bodies (Figure 6J). The anterior zone also displayed variable results in terms of AR expression according to each of the three animals sampled. Although all three samples exhibited AR positivity in more than 50% of cells, the intensity of expression was moderate to strong in koala #1 (Figure 6M), and moderate in koalas #2 (Figure 6P) and #3 (Figure 6S).

#### Central prostate

In the central zone, 10–50% of cells were strongly p63 positive and formed a discontinuous cell layer. A higher number of p63-positive cells were observed in the central zone, compared with the anterior and posterior zones (Figure 6B). Less than 10% of basal cells expressed cytoplasmic CK14 with moderate to strong intensity of staining (Figure 6E). More than 50% of cells were strongly positive for CK8/18 (Figure 6H). The central zone showed 10–20% of cells with mild cytoplasmic expression of PSA (Figure 6K). The central zone demonstrated variable results with the luminal cell marker AR across the samples from each of the three koalas. Koala #1 was positive for AR in more than 50% of cells with a strong intensity of staining (Figure 6N). In koala #2, AR was expressed in approximately 10% of cells with a mild level of staining (Figure 6Q). Koala #3 demonstrated AR in 10–50% of cells with moderate levels of intensity (Figure 6T).

#### Posterior prostate

P63 was expressed in 10–50% of the cells of the posterior zone, showing a strong intensity of nuclear staining (Figure 6C). P63-positive cells formed a discontinuous layer close to the basement membrane. Less than 1% of basal cells were positive for CK14 (Figure 6F) with mild to moderate cytoplasmic staining, with large portions of this segment being consistently CK14 negative. The luminal marker CK8/18 was positive in more than 50% of cells, with a strong intensity of cytoplasmic expression (Figure 6I). More than 50% of luminal cells were positive for PSA, with a mild cytoplasmic staining (Figure 6L). The posterior zone demonstrated variable AR expression across the three koalas: in koala #1 more than 50% of cells expressed AR with a strong intensity of staining (Figure 6O), whereas 10–50% of cells in koala #2 expressed AR with mild intensity of staining (Figure 6R), and more than 50% of cells in koala #3 were AR positive with a strong intensity of staining (Figure 6V).

#### Miscellaneous findings

**Figure 7:**
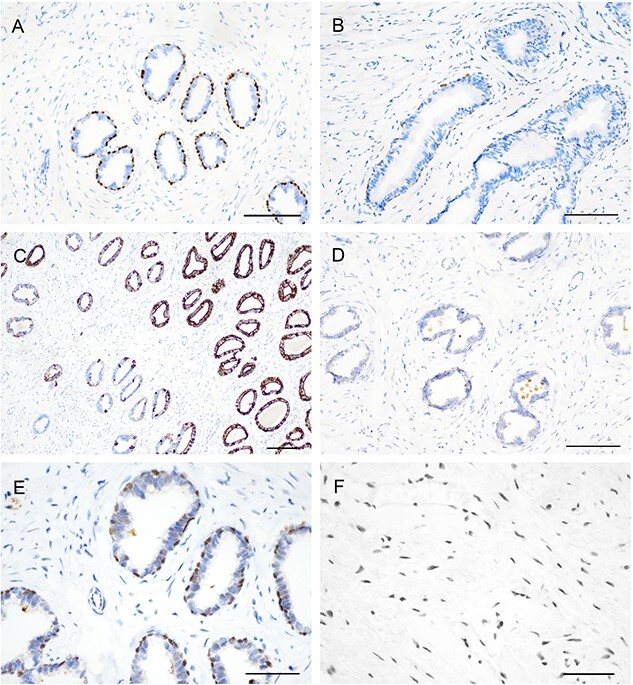
Immunohistochemical features of the periurethral ducts and stroma of the koala’s prostate. (**A**) Continuous layer of p63-positive basal cells (bar = 100 μm). (**B**) Rare scattering of CK14 positive basal cells (bar = 100 μm). (**C**) Strong expression of CK18 in more than 50% of luminal cells (bar = 100 μm). (**D**) Mild AR staining in 20–50% of luminal cells (bar = 100 μm). (**E**) Mild cytoplasmic staining for PSA (bar = 50 μm). (**F**) AR-positive cells throughout the stroma (bar = 50 μm).

Results of the immunohistochemical analysis of the koala periurethral ducts are illustrated in Figure 7. The periurethral ducts were characterized by an almost continuous layer of p63-positive basal cells (Figure 7A) and rare and randomly scattered CK14-positive basal cells (Figure 7B). More than 50% of luminal cells exhibited strong CK18 staining, with higher amounts of expression toward the periphery (Figure 7C). There was mild AR staining in 20–50% of luminal cells (Figure 7D) and mild cytoplasmic staining for PSA only in the ducts close to the glandular structures with a consistently negative staining of the ducts in close proximity to the prostatic urethra (Figure 7E). AR-positive stromal cells were observed throughout the prostate (Figure 7F).

## Discussion

The three segments of the koala prostate were clearly distinguishable based on specific histological features and secretory composition, and which was in general agreement with previous studies conducted by Temple-Smith and Taggart [18] and Pagliarani [8]. The Alcian blue and PAS staining conducted in this study provided additional information on the biochemistry of the respective secretions from each segment, further suggesting that this organ may have multiple roles in the production of seminal fluid components that will require further molecular analysis (proteomic) analysis. This study also represents the first immunohistochemical investigation of the prostate for any marsupial. The cell markers (P63, CK14, CK8/18, AR, PSA) used in this study displayed adequate reactivity in line with the positive and negative controls to assume that the epithelium of the male koala prostate gland contains two well-defined cell populations, a basal (proliferating) cohort and luminal (secretory) cells. Furthermore, the ratio of basal to luminal cells differed between the posterior, central and anterior segments as described in the following sections, further reinforcing the delineation of the segmentation.

In this study, the posterior prostate, the largest of the zones, contained more glandular tissue and significantly thinner collagen fibers. The secretory material was granular in appearance as previously described by Temple-Smith and Taggart [18] and Pagliarani [8]. While Pagliarani [8] found that PAS-positive material largely accumulated in the posterior prostate, the current study revealed only a small number of intermittent PAS-positive globules is this zone. These differences may be attributed to seasonal differences in the production of PAS-positive secretory materials. However, it is worthwhile noting that the samples in Pagliarani’s [8] study had an active *Chlamydia* infection, and it is possible that the infection may also have altered the composition of the secretions, particularly as the koala accessory glands (prostate and bulbourethral glands) have been theorized to be a potential reservoir for *Chlamydia* infection [7]. With respect to the epithelial cell populations, given that the posterior prostate strongly expressed p63, with 10–50% of this cell population positive, it is likely that the posterior zone primarily harbors the proliferative compartment of the koala prostate.

The variability of the AR expression with respect to each koala examined may be interpreted as a reproductive tract that was in differing states of reproductive activity. Koala #2, which exhibited lower levels of AR expression and a milder level of staining, was euthanized in May 2022. Tissue from koala #3 was collected in July and displayed a strong intensity of AR expression in 50% of cells. The sample from koala #1, which was euthanized in September 2020, demonstrated more than 50% of cells positive and with strong intensity of staining. These observations are consistent with an increase in the expression of AR both in number and intensity as the male koala approaches the breeding season.

AR-positive cells were also present throughout the stroma, which is common also in human prostatic tissue [19]. As a key reproductive hormone, androgens have a role in prostate physiology, and stromal AR, in particular, is responsible for prostate organogenesis and epithelial cell growth [19]. Given androgen concentrations have been found to increase significantly in koalas undergoing sexual maturation and then remain high, with seasonal increases around breeding season [20, 21], it is likely that androgen and AR have a similar functional role in the koala prostate. It is understandable then that there is a corresponding higher expression of AR, given its role in binding androgenic hormones [22].

As previously noted by Temple-Smith and Taggart [18] and Pagliarani [8], the central segment of the prostate was the smallest of the three segments. It contained more stroma and less glandular tissue, and the collagen fibers were comparatively thicker. The secretions were homogenous in nature and contained PAS- and Alcian blue-negative material. Like the posterior zone, there was a strong indication of p63 in approximately half the cells, which infers that this zone is also primarily responsible for cell proliferation. PSA expression was significantly lower in the central zone when compared with posterior and anterior zones, suggesting that the central prostate likely has a much smaller role in the production of secretory material. However, this zone also demonstrated an increase in the quantity and intensity of AR luminal cell marker that was positively correlated with koala approaching the breeding season. Given the distinctively homogenous nature of the secretory material, despite its minor role, the central segment may still contribute with specific components to seminal fluid.

The anterior prostate possessed the thinnest collagen fibers of all three zones. It contained more glandular tissue and like the posterior, it was much larger than the central zone. PAS-positive granules were present intermittently in the epithelial cells and the secretory material was determined to be Alcian blue negative. The current study showed the anterior zone contained the majority of PAS-positive secretory material, contrary to previous research by Pagliarani [8] which found the highest volumes in the posterior region. Glycogen, as indicated through PAS staining, has been cited as an energy substrate present in the prostate of other marsupials, including members of the Dasyuridae family (small carnivorous marsupials) [23], the American opossum (*Didelphis virginiana*), and wombats (*Vombatidae*) [24, 2]. Given the reoccurring presence of PAS positivity in studies of the koala’s prostate, it is possible that the koala also uses glycogen as a source of energy for spermatozoa. The variation noted between this study and that of Pagliarani’s [8] with respect to PAS accumulation requires further investigation, but it may reflect seasonal physiological, or even pathological differences. Comparative studies focusing on the effects of seasonality and the changes brought about by chlamydiosis would be useful for defining the normal composition of the glycogen deposits. A biochemical analysis of koala semen will also shed light on the role of glycogen in supporting sperm physiology, particularly with respect to koala sperm cell’s ability to survive prolonged periods when chilled [25].

The epithelium of the anterior zone was highly positive for PSA, with strong expression in more than 50% of cells. Luminal cell markers expressed in more than 50% of cells in this zone. This strongly suggests that the anterior prostate houses much of the secretory function of the prostate, whereas proliferation is a minor component. Furthermore, the anterior prostatic tissue samples from all three animals showed AR expression in more than 50% of cells, the intensity of which increased as the collection period approached breeding season.

In line with previous studies, the anterior zone was filled with large volumes of globular secretions [8, 26]. In marsupials, globular prostatic bodies have been found in the seminal fluid of brushtail possums (*Trichosurus vulpecula*) [27], Tasmanian devils (*Sarcophilus harrisii*) [28], red kangaroos (*Macropus rufus*), eastern gray kangaroos (*Macropus giganteus*) [27], and koalas [26]. Koalas, in particular, are known to possess seminal fluid that is composed mainly of prostatic bodies [26]. It is unclear, as yet, whether these prostatic bodies can be regarded as “true prostasomes” such as those that are present in human seminal fluid; nevertheless, koala prostatic bodies appear at least to be the closest in comparison to human prostasomes in terms of their morphology and proteomics [3].

Specific markers have been used to determine which vesicles are prostasomes by their presence on both prostate epithelial cells and prostasomes and one of these markers is PSA [29]. The current study confirmed PSA expression in exosomes in the anterior zone of the koala prostate, which could be considered prostasome-like material. In human prostatic fluid, these bodies are hypothesized to have a significant role in fertility, including antimicrobial activity, aiding coagulation [30], promoting motility [31], improving timing of the acrosome reaction [32], preservation of membrane integrity, and prolonged sperm survival [33]. Johnston and Holt [6] and more recently Skerrett-Byrne et al. [3] have reported that the koala displays the longest known viability of mammalian germ cells in chilled storage, lasting up to 42 days [34]. Given that prostasomes have a key role in sperm survival, confirming these bodies in the koala are the same vesicles that exist in humans could have far-reaching applications for assisted reproduction in both species.

As previously described [18, 8], the koala’s prostate was determined to be encapsulated by an outer layer of connective tissue and a thick inner layer of smooth muscle cells. In two of the three koalas, there were large amounts of pale basophilic material interspersed in the stromal connective tissue. This myxoid matrix mainly accumulated around the prostatic urethra and was confirmed to be Alcian blue positive. This is the first study to detect mucins of this nature in the stroma of the koala’s prostate gland and the first to describe any presence of Alcian blue-positive material in the gland. The prostates that were positive for acidic mucins were collected from koalas (#1 and #3) euthanized in July and early September. This is immediately prior to the breeding season, documented to be from September to December in Southeast Queensland koalas [35, 36]. The sample without mucous material was collected in May (#2). The highest concentration of testosterone in koalas also occurs immediately before the breeding season [35] coinciding with the highest dispersal of male koalas in July and August [37]. Given these samples were collected during this same period, the presence of acidic mucins is likely associated with elevated androgen levels. As only three koala prostates were sampled, it was not possible to compare prostates before, during, and after the breeding season.

The expression of basal cell marker, CK14, varies in prostatic basal cells in different species and is, therefore, not reliable for confirming an accurate count of basal cells. In the koala, CK14-expressing cells were present but generally scarce, similar to the incidence and pattern of expression seen in canines [38]. This is unlike the human prostate where most of the cells express CK14 [39]. Conversely, p63 is consistently expressed in prostate epithelial basal cells across all species [40] and is therefore a more reliable marker to confirm basal cell numbers. Expression of this antibody in the koala prostate confirmed 20–30% of cells to be proliferative cells. Without quantitative analysis, it is difficult to determine exactly the ratio of basal to luminal cells; however, luminal markers confirmed that the large majority of epithelial cells were secretory. The prostate gland of humans also displays a high number of luminal cells (80–90%) to basal cells (10–20%) [41], so that the overall composition and immunophenotype of humans and koalas is markedly similar.

The main point of distinction between the basal and luminal cell populations in the human prostate and the koala is the organization of the cell layers. In the koala, there is an almost contiguous layer of basal cells in the ductal epithelium, yet there are gaps in the continuity of the layer of basal cells in the glandular acini. Contrary to this, the human prostatic acini have a continuous layer of basal cells [42]. This discontinuous arrangement in the koala is most likely because of the interposition of secretory cells that have direct contact with the basement membrane and is similar to that found in the canine prostate [16].

Research on the cell ratio across the different zones of the human prostate is inconsistent. Some studies state that there is no difference in basal and luminal cytokeratin markers between the three zones [43]. However, recent research has suggested that there are notably more luminal cells in the peripheral zone, more basal proliferating cells in the transitional zone and very few proliferating cells in the central zone [9]. It is possible, therefore, that both the koala prostate gland and the human prostate gland have a functional compartmentalization of the prostate with sections showing a more significant role in the production of secretions and others, a greater involvement in the proliferation and cellular turnover. We suggest that the central zone of the koala prostate may be homologous to the transitional zone of the human prostatic gland and the anterior zone may be akin to the human peripheral zone. Given that zonal architecture is not present in all animals, this provides further support that the koala may be a useful a model for studying prostate biology in humans.

## Conclusion

This study has confirmed the zonal architecture of the koala prostate gland. Using histology and immunohistochemical markers, anterior, central, and posterior zones were all clearly defined in terms of their histological architecture, relative cellular composition, and apocrine secretion. Furthermore, immunohistochemistry confirmed that the epithelial cell types vary in ratios across each zone, with the anterior prostate containing the highest amounts of secretory cells and activity. These observations provide support to the hypothesis that the functional role of the prostate’s zones is likely to produce varying components of the seminal fluid. We also established that many of the antibodies used in typical prostate gland IHC were also effective in koala tissue. It is clear that the koala epithelial tissue contains two distinct cell populations: basal proliferative and luminal secretory cells. Evidence was also provided for the theory that exosomes released by the anterior zone of the koala prostate are structurally and biochemically similar to that of human prostasomes. This is the first description of PSA-positive prostasome-like bodies for a marsupial species. To confirm that these are indeed the same globular bodies present in humans, further investigations, including gene expression and TEM, would be required. Confirming the presence of true prostasomes in the koala would be the basis for further research into their role in male fertility (e.g., prolonged sperm survival) and their use as a model for prostasome research in humans. Although only based on three animals, it was also shown that there is an increase in the activity of luminal cells as the male koala prepares for breeding season.

Given this research supports the histological division of the koala prostate, future research should now focus on a more detailed understanding of the molecular and functional significance of this zonal segmentation and whether the koala represents an example of an ontological stage in the evolutionary differentiation of the male therian accessory glands. In particular, proteomic analysis would be useful to understanding the function of each of these fluids. In addition, comparative studies of prostate glandular activity throughout the year would be valuable in confirming whether the changes observed in this study were reflective of reproductive seasonality and changes in semen quality. Finally, electron microscopy of pathology-free and prostate glands infected with chlamydiosis would provide evidence as to whether the prostate gland, more particularly exosomes (presumptive prostasomes), might also be a vehicle for the transmission of *Chlamydia*.

## Data Availability

The data underlying this article are available in the article and in its online supplementary material.

## Declaration of originality

This research is the original work of all authors.

## References

[1] Dawson TJ, Finch E, Freedman L, Hume ID, Renfree MB, Temple-Smith PD. Chapter 17: Morphology and physiology of the Metatheria. In: Walton DW, Richardson BJ (eds.), Fauna of Australia, vol. 1B. Canberra: AGPS Canberra; 1989: 53–64.

[2] Tyndale-Biscoe H, Renfree M. Monographs on Marsupial Biology; Reproductive Physiology of Marsupials. Melbourne: Cambridge University Press; 1987: 124–171.

[3] Skerrett-Byrne DA, Anderson AL, Hulse L, Wass C, Dun MD, Bromfield EG, De Iuliis GN, Pyne M, Nicolson V, Johnston SD, Nixon B. Proteomic analysis of koala (*Phascolarctos cinereus*) spermatozoa and prostatic bodies. Proteomics 2021; 21:e2100067. 34411425 10.1002/pmic.202100067

[4] Johnston SD, O'Callaghan PO, Nilsson K, Tzipori G, Curlewis J. Semen-induced luteal phase and identification of a LH surge in the koala (*Phascolarctos cinereus*). Reprod Fertil 2004; 128:629–634. 10.1530/rep.1.00300 15509709

[5] Woinarski JCZ, Burbidge AA, Harrison PL. The Action Plan for Australian Mammals. Collingwood: CSIRO Publishing; 2012.

[6] Johnston SD, Holt WV. The koala (*Phascolarctos cinereus*): a case study in the development of reproductive technology in a marsupial. Adv Exp Med Biol 2014; 753:171–203. 25091911 10.1007/978-1-4939-0820-2_9

[7] Palmieri C, Hulse L, Pagliarani S, Larkin R, Higgins DP, Beagley K, Johnston SD. *Chlamydia pecorum* infection in the male reproductive system of koalas (*Phascolarctos cinereus*). Vet Pathol 2019; 56:300–306. 30381016 10.1177/0300985818806963

[8] Pagliarani S . Further insights into the pathogenesis of Chlamydiosis and characterisation of immune cells in the reproductive tract of koalas (Phascolarctos cinereus). PhD thesis. Gatton: University of Queensland; 2021.

[9] Ali A, Du Feu A, Oliveira P, Choudhury A, Bristow RG, Baena E. Prostate zones and cancer: lost in transition? Nat Rev Urol 2022; 19:101–115. 34667303 10.1038/s41585-021-00524-7

[10] Rodger JC, Hughes RL. Studies of the accessory glands of male marsupials. Aust J Zool 1973; 21:303–320.

[11] Urabe F, Kosaka N, Asano K, Egawa S, Ochiya T. Chapter 5 - Physiological and pathological functions of prostasomes: from basic research to clinical application. In: Edelstein L, Smythies J, Quesenberry P, Noble D (eds.), Exosomes. Amsterdam: Elsevier; 2020: 101–121.

[12] Hulse LS, Hickey DK, Mitchell CM, Ellis W, Beagley K, Johnston S. The development and application of two multiplex real time PCR assays for the detection and speciation of bacterial pathogens in the koala (*Phascolarctos cinereus*). J Vet Diagn Invest 2018; 30:523–529. 29629645 10.1177/1040638718770490 PMC6505923

[13] Hulse LS, McDonald S, Johnston SD, Beagley KW. Rapid point-of-care diagnostics for the detection of *Chlamydia pecorum* in koalas (*Phascolarctos cinereus*) using loop-mediated isothermal amplification without nucleic acid purification. Microbiology 2019; 8:e916. 10.1002/mbo3.916 PMC6925175 31419041

[14] Bedossa P, Paradis V, Zucman-Rossi J. 2 - Cellular and molecular techniques. In: Burt AD, Ferrell LD, Hübscher SG (eds.), Macsween’s Pathology of the Liver, 7th ed. Amsterdam: Elsevier; 2018: 88–110.

[15] Lai M, Lü B. 3 - Tissue preparation for microscopy and histology. In: Pawliszyn J, Bayona J, Dugo P, Le CX, Lee HK, Li XF, Lord H (eds.), Comprehensive Sampling and Sample Preparation, 1st ed. Cambridge: Academic Press; 2012: 53–93.

[16] Palmieri C, Fonseca-Alves CE, Laufer-Amorim R. A review on canine and feline prostate pathology. Front Vet Sci 2022; 9:e881232. 10.3389/fvets.2022.881232 PMC9201985 35720846

[17] Altschul SF, Gish W, Miller W, Myers EW, Lipman DJ. Basic local alignment search tool. J Mol Biol 1990; 215:403–410. 2231712 10.1016/S0022-2836(05)80360-2

[18] Temple-Smith PD, Taggart DA. On the male generative organs of the koala (*Phascolarctos cinereus*): an update. In: Lee AK, Handasyde KA, Sanson GD (eds.), Biology of the Koala. Chipping North: Surrey Beatty & Sons; 1990: 33–54.

[19] Liu Y, Wang J, Horton C, Yu C, Knudsen B, Stefanson J, Hu K, Stefanson O, Green J, Guo C, Xie Q, Wang ZA. Stromal AR inhibits prostate tumor progression by restraining secretory luminal epithelial cells. Cell Rep 2022; 39:e110848. 10.1016/j.celrep.2022.110848 PMC9175887 35613593

[20] Cleva GM, Stone GM, Dickens RK. Variation in reproductive parameters in the captive male koala (*Phascolarctos cinereus*). Reprod Fertil Dev 1994; 6:713–719. 7624512 10.1071/rd9940713

[21] Kusuda S, Hashikawa H, Takeda M, Ito H, Goto A, Oguchi J, Doi O. Season- and age-related reproductive changes based on fecal androgen concentrations in male koalas *Phascolarctos cinereus*. J Reprod Dev 2013; 59:308–313. 23502854 10.1262/jrd.2012-188 PMC3934141

[22] Roy AK, Lavrovsky Y, Song CS, Chen S, Jung MH, Velu NK, Bi BY, Chatterjee B. Regulation of androgen action. In: Litwack G (ed.), Vitamins & Hormones, vol. 55. Amsterdam: Elsevier; 1998: 309–352. 10.1016/s0083-6729(08)60938-3 9949684

[23] Rodger JC, White IG. Glycogen not N-acetylglucosamine the prostatic carbohydrate of three Australian and American marsupials, and patterns of these sugars in marsupialia. Comp Biochem Physiol B 1980; 67:109–111.

[24] Brooks DE, Gaughwin MD, Mann M, Mann T. Structural and biochemical characteristics of the male accessory organs of reproduction in the hairy-nosed wombats (*Lasiorhinus latifrons*). J Zool 1978; 201:191–207. 10.1098/rspb.1978.0040 27799

[25] Johnston SD, McGowan MR, Phillips NJ, O'Callaghan P. Optimal physico-chemical conditions for the manipulation and short-term preservation of koala (*Phascolarctos cinereus*) spermatozoa. Reprod Fertil 2000; 118:273–281. 10.1530/jrf.0.1180273 10864791

[26] Wildt DE, Maximilian B, O'Brien SJ, Murray ND, Taylor A, Graves J. Semen characteristics in free-living koalas (*Phascolarctos cinereus*). Reprod Fertil 1991; 92:99–107. 10.1530/jrf.0.0920099 2056498

[27] Rodger JC and White IG. The collection handling and some properties of marsupial semen. In: Watson PE (ed.) The Artificial Breeding of Non-Domestic Animals, London: Academic Press; 1978:289–301.

[28] Keeley T, Harris M, McGreevy PD, Hudson D, Obrien JK. Development and evaluation of electroejaculation techniques in the Tasmanian devil (Sarcophilus harrisii). Reprod Fertil Dev 2012; 24:1008–1018. 22935162 10.1071/RD12022

[29] Aalberts M, Stout T, Stoorvogel W. Prostasomes: extracellular vesicles from the prostate. Reproduction 2014; 147:R1–R14. 24149515 10.1530/REP-13-0358

[30] Sahlén G . Formation, storage and secretion of prostasomes in benign and malignant cells and their immunogenicity in prostate cancer patients. PhD thesis. Uppsala: Uppsala University; 2007.

[31] Carlsson L, Ronquist G, Stridsberg M, Johansson L. Motility stimulant effects of prostasome inclusion in swim-up medium on cryopreserved human spermatozoa. Arch Androl 2009; 38:215–221. 10.3109/01485019708994880 9140618

[32] Kravets FG, Lee J, Singh B, Trocchia A, Pentyala SN, Khan SA. Prostasomes: current concepts. Prostate 2000; 43:169–174. 10797491 10.1002/(sici)1097-0045(20000515)43:3<169::aid-pros2>3.0.co;2-d

[33] Arienti G, Carlini E, Nicolucci A, Cosmi V, Santi F, Palmerini CA. The motility of human spermatozoa as influenced by prostasomes at various pH levels. Biol Cell 1999; 91:51–54. 10321022

[34] Johnston SD, McGowan MR, Phillips NJ, O'Callaghan P. Optimal physico-chemical conditions for the manipulation and short-term preservation of koala (*Phascolarctos cinereus*) spermatozoa. Reprod Fertil 2000; 118:273–281. 10.1530/jrf.0.1180273 10864791

[35] Allen CD, de Villiers DL, Manning BD, Dique DS, Burridge M, Chafer ML, Nicolson VN, Jago SC, McKinnon AJ, Booth RJ, McKee JJ, Pyne MJ, et al. Seasonal reproduction in wild and captive male koala (*Phascolarctos cinereus*) populations in south-east Queensland. Reprod Fertil Dev 2010; 22:695–709. 20353729 10.1071/RD09113

[36] Ellis W, FitzGibbon S, Pye G, Whipple B, Barth B, Johnston S, Seddon J, Melzer A, Higgins D, Bercovitch F. The role of bioacoustic signals in koala sexual selection: insights from seasonal patterns of associations revealed with GPS-proximity units. PloS One 2015; 10:e0130657. 26154295 10.1371/journal.pone.0130657 PMC4496073

[37] Dique DS, Thompson J, Preece HJ, Villiers DL, de Carrick FN. Dispersal patterns in a regional koala population in south-east Queensland. Wildl Res 2003; 30:281–290.

[38] de Brot S, Lothion-Roy J, Grau-Roma L, White E, Guscetti F, Rubin MA, Mongan MP. Histological and immunohistochemical investigation of canine prostate carcinoma with identification of common intraductal carcinoma component. Vet Comp Oncol 2022; 20:38–49. 33963663 10.1111/vco.12704 PMC9292867

[39] Wang Y, Hayward S, Cao M, Thayer K, Cunha G. Cell differentiation lineage in the prostate. Differentiation 2001; 68:270–279. 11776479 10.1046/j.1432-0436.2001.680414.x

[40] Signoretti S, Loda M. Defining cell lineages in the prostate epithelium. Cell Cycle 2006; 5:138–141. 16357539 10.4161/cc.5.2.2340

[41] Humphrey PA . Prostate Pathology. Chicago: ASCP; 2003.

[42] Rebello RJ, Oing C, Knudsen KE, Loeb S, Johnson DC, Reiter RE, Gillessen S, Van der Kwast T, Bristow RG. Prostate cancer. Nat Rev Dis Prim 2021; 7:9. 33542230 10.1038/s41572-020-00243-0

[43] Laczkó I, Hudson DL, Freeman A, Feneley MR, Masters JR. Comparison of the zones of the human prostate with the seminal vesicle: morphology, immunohistochemistry, and cell kinetics. Prostate 2005; 62:260–266. 15389778 10.1002/pros.20149

[44] Young AH . On the male generative organs of the koala (*Phascolarctos cinereus*). J Anat Physiol 1879; 13:305–331. PMC1309851 17231260

[45] Larkin R, Palmieri C, Oishi M, Hulse L, Johnston SD. Ultrasonographic assessment of the male koala (*Phascolarctos cinereus*) reproductive tract. Res Vet Sci 2018; 117:219–223. 29306150 10.1016/j.rvsc.2017.12.019

